# Early tracheostomy in severe head injuries at a rural center

**DOI:** 10.4103/0974-2700.44687

**Published:** 2009

**Authors:** Amit Agrawal, S R Joharapurkar, K B Golhar, V V Shahapurkar

**Affiliations:** Department of Surgery, Datta Meghe Institute of Medical Sciences, Sawangi (Meghe), Wardha, India

Sir,

Neurosurgical patients including patients with severe head injury are at risk of developing respiratory complications, adversely affecting outcome and survival.[1] Majority of these patients with severe head injury may need ventilatory support.[2] Tracheostomy in this group of patients is associated with fewer risks than prolonged endotracheal intubation,[2,3] decreases total days of mechanical ventilation,[4] resulting in a shorter ICU stay.[2] We retrospectively reviewed our experience with 20 patients (form 1st April 2007 to 31st March 2008) of severe head injury in whom early tracheostomy was performed. Apart from the role of tracheostomy we discuss the social aspects of such procedure in reference to the rural social milieu. After getting the ethical approval clinical records of the entire 20 patient were reviewed. These 20 cases of severe head injury (GCS score of less than 8) underwent early trachesotomy (within 72 hours). All patients were managed as per ATLS protocol. If required, endotracheal intubation was performed at any time before perform tracheostomy. Out of 20, 13 patents expired because of the severity of head injury. In survived patients, mean ventilator demand was reduced. One patient is able to attend his duties and others are doing well although improving but with significant cognitive deficits [Table 1]. The role of trachesotomy in severe head injury is well established.[5] Traumatic brain injury patients presenting with a GCS <8, an ISS >25, and ventilator days >7 are more likely to be a candidate for tracheostomy.[6] In developing countries where the intensive care facilities are scarce and may not be easily available even at tertiary referral centers, many critical patients have to be managed in high dependency cubicles in the ward, often with inadequately trained nursing staff and equipment to monitor them.[7] In such circumstances, early tracheostomy has been shown to be beneficial in normalizing systemic physiological parameters in patient with severe head injury.[7] It becomes more difficult in distant rural areas of developing countries to provide such advanced care, however in our initial experience we find that early tracheostomy helped us to mange the survived patients better. However, there were unanswered queries not related to the scientific issues but were related to social aspects. After initial hurdles, almost all the relatives were very co-operative and participated in patient care. In spite of their confidence in taking care of the patient (nasogastric feeding, position change, support for day-to-day activities, etc.) none of them wanted to take the patient (once the condition is stabilized) either home or to any other health care center while the patient was on tracheostomy. Many of relatives taking care of the patients were not well educated but very affectionate to the patients. We find the main worries were suctioning and change of tracheostomy tube. Once the tracheostomy tube was removed, it was easy for us to discharge the patient. There were options to do suctioning either with electric suction machine or paddle driven suction machine. We could not advise electric suction machine as there was no regular electricity supply at their home. While advising paddle driven suction machine we realized that we never used that suction machine or demonstrated the functioning of the machine as working with it was not very easy. In summary, with this simple example we would like to highlight although we have convincing scientific evidence that this particular mode of therapy can help, however while conducing any study or formulating a treatment plan there is need to associate it with social requirements particularly expectations from the relatives and expectations and limitations of the relatives.

**Table 1:** Summary of results (n=20)

Outcome	Number
Patients survived	7 (ventilator demand - mean 5.4 days)
One patient (teacher)	Returned to duty
Improving but having cognitive deficits	6 (Able to do day to day activities-3)
Tracheal stenosis	1 (conservative management)
Mortality	13

## References

[1] Rozet I, Domino KB (2007). Respiratory care. Best Pract Res Clin Anaesthesiol.

[2] Ahmed N, Kuo YH (2007). Early versus late tracheostomy in patients with severe traumatic head injury. Surg Infect (Larchmt).

[3] Goettler CE, Fugo JR, Bard MR, Newell MA, Sagraves SG, Toschlog EA (2006). Predicting the need for early tracheostomy: A multifactorial analysis of 992 intubated trauma patients. J Trauma.

[4] Bouderka MA, Fakhir B, Bouaggad A, Hmamouchi B, Hamoudi D, Harti A (2004). Early tracheostomy versus prolonged endotracheal intubation in severe head injury. J Trauma.

[5] Major KM, Hui T, Wilson MT, Gaon MD, Shabot MM, Margulies DR (2003). Objective indications for early tracheostomy after blunt head trauma. Am J Surg.

[6] Gurkin SA, Parikshak M, Kralovich KA, Horst HM, Agarwal V, Payne N (2002). Indicators for tracheostomy in patients with traumatic brain injury. Am Surg.

[7] Chintamani, Khanna J, Singh JP, Kulshreshtha P, Kalra P, Priyambada B (2005). Early tracheostomy in closed head injuries: Experience at a tertiary center in a developing country--a prospective study. BMC Emerg Med.

